# Effects of four types of natural bait on water quality, feeding, growth, and antioxidant enzyme activity of *Monopterus albus* in a recirculating aquaculture system

**DOI:** 10.3389/fphys.2024.1403391

**Published:** 2024-06-13

**Authors:** Quan Yuan, Chengcheng Wu, Hang Yang, Weiwei Lv, Weiwei Huang, Qinghua Zhang, Wenzong Zhou

**Affiliations:** ^1^ Eco-Environmental Protection Research Institute, Shanghai Academy of Agricultural Sciences, Shanghai, China; ^2^ National Agricultural Experimental Station for Agricultural Environment, Shanghai, China; ^3^ Key Laboratory of Exploration and Utilization of Aquatic Genetic Resources, Ministry of Education, Shanghai Ocean University, Shanghai, China

**Keywords:** Asian swamp eel, *Tenebrio molitor*, earthworm, housefly maggot, recirculating aquaculture system

## Abstract

Monopterus albus is one of China’s renowned and superior aquaculture species, with its seedlings mainly sourced from wild capture. One of the bottlenecks in *M. albus* aquaculture is the high mortality rate and low feeding initiation rate from stocking wild fry to the initiation of feeding. In production, trash fish is commonly used to wean *M. albus* juveniles onto feeding. In this study, we introduced three other natural feeds, earthworms (EW), yellow mealworms (YMW), and fly maggots (FM), with frozen trash fish (TF) serving as the control group, to evaluate the effects of these four natural feeds on the survival rate, feeding initiation, antioxidant enzymes activity, and body composition of *M. albus* juveniles under recirculating water aquaculture conditions. The experiment comprised four treatments, each with three replicates. Each replicate consisted of stocking 150 *M. albus* juveniles weighing 10.02 ± 0.89 g in size, raised for 5 weeks. The survival rate of the YMW group was 73.33%–85.33%, which was significantly higher than that of the other three bait groups (*p* < 0.05). The four bait groups showed no significant differences in final body weight and specific growth rate (SGR) (*p* > 0.05). The EW group showed the highest final body weight, with an average SGR of 2.73, whereas the YMW group had an average SGR of 1.87. The average daily feeding amount was significantly higher in EW and YMW groups than in the other two groups (*p* < 0.05). The percentage of feeding amount to fish weight in the EW group reached 7.3% in the fifth week. After 5 weeks of cultivation, NO_2_^−^-N content was significantly higher in the waters of the TF and EW groups than in the waters of the FM and YMW groups (*p* < 0.05), there was no significant difference in TAN content among the treatment groups (*p* > 0.05). Liver malondialdehyde content was significantly higher in the TF group than in the other bait groups (*p* < 0.05). GSH-Px activity was significantly higher in the EW group than in the FM group and YMW group. No significant differences in SOD and CAT activity and T-AOC were observed among the bait groups (*p* > 0.05). The increase in crude protein content was significantly higher in the TF group than in the FM group, but the increase in crude ash content was significantly lower in the TFgroup. In conclusion, *Tenebrio molitor* could potentially serve as one of the alternative feeds during the initial stages of *M. albus* juveniles stocking.

## 1 Introduction

The Asian swamp eel, *Monopterus albus* (Zuiew, 1793), is one of the most economically important freshwater fish species in China and other Asian countries (Zhou et al., 2002), namely, Cambodia, Singapore, Thailand, and Vietnam (Pedersen et al., 2014). Its production were close to 334, 215 tons, and the annual output value was nearly 23 billion yuan in 2022 (The Fisheries Bureau of Ministry of Agriculture, 2023). In China, three major farming regions with large-scale culture of *M. albus* are Hubei, Jiangxi, and Anhui, which account for 154, 279 tons, 89, 943 tons, and 32, 880 tons (The Fisheries Bureau of Ministry of Agriculture, 2023). Currently, pond cage culture is the main method for *M. albus* culture, and other methods are paddy field culture and soil pond culture.

Since the 21st century, recirculating aquaculture, as a new type of aquaculture mode, has addressed the drawbacks of traditional aquaculture methods, reduced the dependence of the aquaculture process on the surrounding water environment, reduced sewage discharge during the aquaculture process, increased yield and quality, and achieved green aquaculture. The application scale of recirculating aquaculture in freshwater fish and shrimp aquaculture is continuously expanding (Ahmed and Turchini, 2021). *M*. *albus* likes to live in groups and can adapt to high-density farming (Luo et al., 2014). In the aquaponics system, the survival rate of *M. albus* can be more than 79% (Portalia et al., 2019). Currently, *M. albus* is cultured at different scales in Hubei and Jiangxi, China, but there is a lack of studies on recirculating aquaculture technology for this species.

Because of the lack of substantial breakthroughs in the artificial reproduction of *M. albus*, the current production of seedlings is mainly relies on wild fishing. Generally, wild seedlings require 2–3 weeks of open domestication after being stocked, and the mortality rate of seedlings is relatively high at this stage (Yuan et al., 2020). Poor nutrition and water quality and parasitic, bacterial, and viral infections can cause serious losses in *M. albus* juveniles under intensive culture conditions (Gao et al., 2016; Shao et al., 2016; Liu et al., 2019; Xia et al., 2019). Therefore, it is important to improve the survival rate and initial feeding success of *M. albus* juveniles during the stocking stage.

In nature, *M. albus* is a nocturnal predator that devours fish, worms, crustaceans, and other small aquatic animals (Zhou et al., 2011; Ma et al., 2014). In traditional practices, *M. albus* is usually fed a commercial formula feed mixed with trash fish or earthworms. However, trash fish can pose serious sanitary risks such as transfer of pathogens or parasites to the eels and water pollution (Morais et al., 2014). Moreover, trash fish have been facing a sharp decline, leading to an unreliable supply and high cost (Tacon and Metian, 2008). Recently, insects have yielded tremendous results as potential replacers of fishmeal in aquafeed, with the black soldier fly (*Hermetia illucens*) and mealworm (*T. molitor*) being the most studied and promising insects (Maulu et al., 2022). In aquaculture, many studies have revealed positive results when black soldier fly meal was used as a substitute for fish meal for many species such as whiteleg shrimp (*Litopenaeus vannamei*) (Richardson et al., 2021) and Nile tilapia (*Oreochromis niloticus*) (Were et al., 2021). *Tenebrio molitor* has shown positive results when utilized in the diets of many aquatic species, such as yellow catfish (*Pelteobagrus fulvidraco*) (Su et al., 2017). Furthermore, *T. molitor* has a relatively high nutritional value, and it is a rich source of essential amino acids (such as methionine), lipids, and fatty acids that vary depending on the developmental stage of the larvae (Shafique et al., 2021).

The aim of this study was to comprehensively evaluate the effects of four types of bait, namely, trash fish, earthworms, fly maggots, and yellow mealworms (*T. molitor*), on the survival rate, feeding, antioxidant enzymes activity, body composition, and aquaculture water quality of *M. albus* juveniles under recirculating aquaculture conditions. The results will provide theoretical support for improving open-mouth domestication technology in the juvenile stage of *M. albus* and important guidance for recirculating aquaculture of *M. albus*.

## 2 Materials and methods

### 2.1 Tank systems

The aquaculture experiment was conducted in cement tanks at the Zhuanghang Comprehensive Experiment Station of Shanghai Academy of Agricultural Sciences. Each cement tank (1.20 m × 1.50 m × 0.80 m) was equipped with an independent freshwater recirculating system and a fish nest as a test unit. The freshwater recirculating system included water pumps, ultraviolet lamps, and filter materials such as biochemical cotton, ceramic rings, and bacterial houses. The fish nest was composed of six S-shaped plastic plates stacked on a flat surface with grooves (1.00 m × 0.50 m × 0.30 m). A hole (diameter, 15–30 cm) passed through the middle of the fish nest. This was the place where *M. albus* was fed at a designated point. The two ends of the *M. albus* nest were fixed with iron wires penetrating through the plastic plates in the cement tank, 1–2 cm away from the water surface.

### 2.2 Experimental design

The following four different bait groups were established: trash fish (TF), earthworms (EW), fly maggots (FM), and yellow mealworms (YMW). In production, trash fish is commonly used to domesticate *M. albus* juveniles onto feeding and served as the control group in this study. Trash fish, earthworms, and fly maggots were purchased from a local market. *Tenebrio molitor* specimens were provided by the Zhuanghang Experimental Station Base of the Shanghai Academy of Agricultural Sciences. The nutritional compositions of the four types of baits are provided in Table 1.

**TABLE 1:** Body composition of the four types of bait. TF, Trash fish; EW, Earthworm; FM, Fly maggot; YMW, yellow mealworm.

Treatment group	Moisture content (%)	Crude protein (%)	Crude fat (g/100 g)	Crude ash (g/100 g)
TF	68.12 ± 0.22	17.78 ± 0.04	10.69 ± 0.05	2.18 ± 0.09
EW	58.03 ± 0.43	7.07 ± 0.09	8.43 ± 0.12	6.21 ± 0.11
FM	68.56 ± 0.13	10.98 ± 0.03	12.13 ± 0.04	2.36 ± 0.05
YMW	68.01 ± 0.07	18.11 ± 0.04	8.84 ± 0.03	5.03 ± 0.04

Each treatment group was set with three replicates, and a total of 12 cement tanks were used in this experiment. On hundred and fifty healthy *M. albus* juveniles (mean ± SD, 10.02 ± 0.89 g) with consistent specifications were stocked in each cement tank, and the juveniles were purchased from Henan Province. The experimental period was 5 weeks.

### 2.3 Culture conditions

The *M. albus* juveniles were purchased and temporarily maintained in a large tank (5.0 m × 3.0 m × 0.5 m). During this period, they were fed with ice-fresh fish once a day. After 2 weeks of temporary cultivation, healthy fish were selected for the experiment. The cement tanks were cleaned and disinfected 1 week before the experiment, and the recirculating water treatment device was inspected. During the experiment, bait was dropped once every afternoon from 3 to 4 p.m. The feeding amount was adjusted according to the feeding situation of *M. albus*, and the daily feeding amount was recorded. Ice-fresh fish was ground into fish paste by using a grinder before each feeding, stirred evenly, and then fed to the *M. albus* juveniles. During the experiment, the recirculating water system worked 24 h and the water was regularly replenished to compensate for evaporation. If dead eels were found, they were promptly removed and recorded.

### 2.4 Sample collection and analysis

At the beginning of the experiment, 3 *M. albus* specimens were randomly collected from each tank, and their weight was measured before they were stored in a refrigerator at −20°C for measuring the nutritional composition. Following the commencement of the experiment, daily surface water temperature readings were recorded using a thermometer, and every 5 days, the dissolved oxygen (DO) and pH levels in each culture tank were measured using a water quality analyzer (HACH HQ40d, United States) *in situ*. Water samples were collected every week, and 250-mL surface water samples from each tank were collected with 300-mL wide-mouth bottles and taken to the laboratory. Total nitrogen (TN), total phosphorus (TP), total ammonia nitrogen (TAN), nitrate nitrogen (NO_3_^−^-N) and nitrite nitrogen (NO_2_^−^-N) levels were measured for each sample within 24 h of collection. TN, TP, TAN, NO_3_^−^-N, and NO_2_^−^-N were measured using the HACH prefabricated reagent, according to the instructions of the *Water Analysis Handbook* (HACH, 2010).

At the end of the experiment, each *M. albus* was weighed, and the number of surviving *M. albus* in each tank was counted. Three *M. albus* specimens were randomly collected from each tank, their body weights were measured, and they were dissected on an ice plate. The liver and muscle tissues were removed, frozen in liquid nitrogen, and stored in a refrigerator at −80°C for testing. Fresh samples of the four bait types were collected and stored in a refrigerator at −20°C for testing.

### 2.5 Growth and feeding indicators

After the feeding experiment, the eels in each cement tank were weighed and counted, and the related growth performance indices of *M. albus* were calculated as follows:

$$\text{Survival rate }\left(\text{SR},\%\right)=\left(\text{number of surviving animals}/\text{total number of animals}\right)\times 100$$

$$\text{Specific growth rate }\left(\text{SGR},\%\right)=\left[\mathrm{Ln}\;\left(\text{final weight}\right)\;–\;\mathrm{Ln}\;\left(\text{initial weight}\right)\right]/\text{days}\times 100$$

$$\text{Feeding percentage }\left(\%\right)\text{ of first week}=\text{daily feeding amount of first week }\left(g\right)/\text{initial body weight }\left(g\right)\times 100\%.$$

$$\text{Feeding percentage }\left(\%\right)\text{ of fifth week}=\text{daily feeding amount of fifth week }\left(g\right)/\text{final body weight }\left(g\right)\times 100\%.$$

### 2.6 Antioxidant enzyme indicies and nutrient composition

The liver tissues were weighed accurately, and pre-cooled saline was added in the ratio of weight (g): volume (mL) = 1:10; the mixture was centrifuged at 2,500 r/min for 10 min in a high-speed centrifuge, and the supernatant was collected for determination. Total antioxidant capacity (T-AOC), malondialdehyde (MDA), glutathione peroxidase (GSH-Px), superoxide dismutase (SOD), and catalase (CAT) in the liver were determined using reagent kits (Jiancheng, Nanjing, China), according to the instructions. The protein content was measure with Coomassie brilliant blue staining (Candiano et al., 2004).

Proximate compositions of the diets and whole body of *M. albus* were determined according to the Association of Official Analytical Chemists (AOAC, 2010).

### 2.7 Statistical analysis

All experimental data were recorded in Microsoft Excel 2013 (Microsoft Co., Redmond, WA, United States), and SPSS 22.0 (IBM Corp., Armonk, NY, United States) software was used for one-way analysis of variance (ANOVA). When the difference was significant (*p* < 0.05), the Tukey’s HSD test was used for multiple comparisons. Origin 22.0 (Electronic Arts Inc., United States) was used for plotting the graphs. The results are expressed as mean ± standard error (SE).

## 3 Results

### 3.1 Survival rate and SGR

**FIGURE 1:**
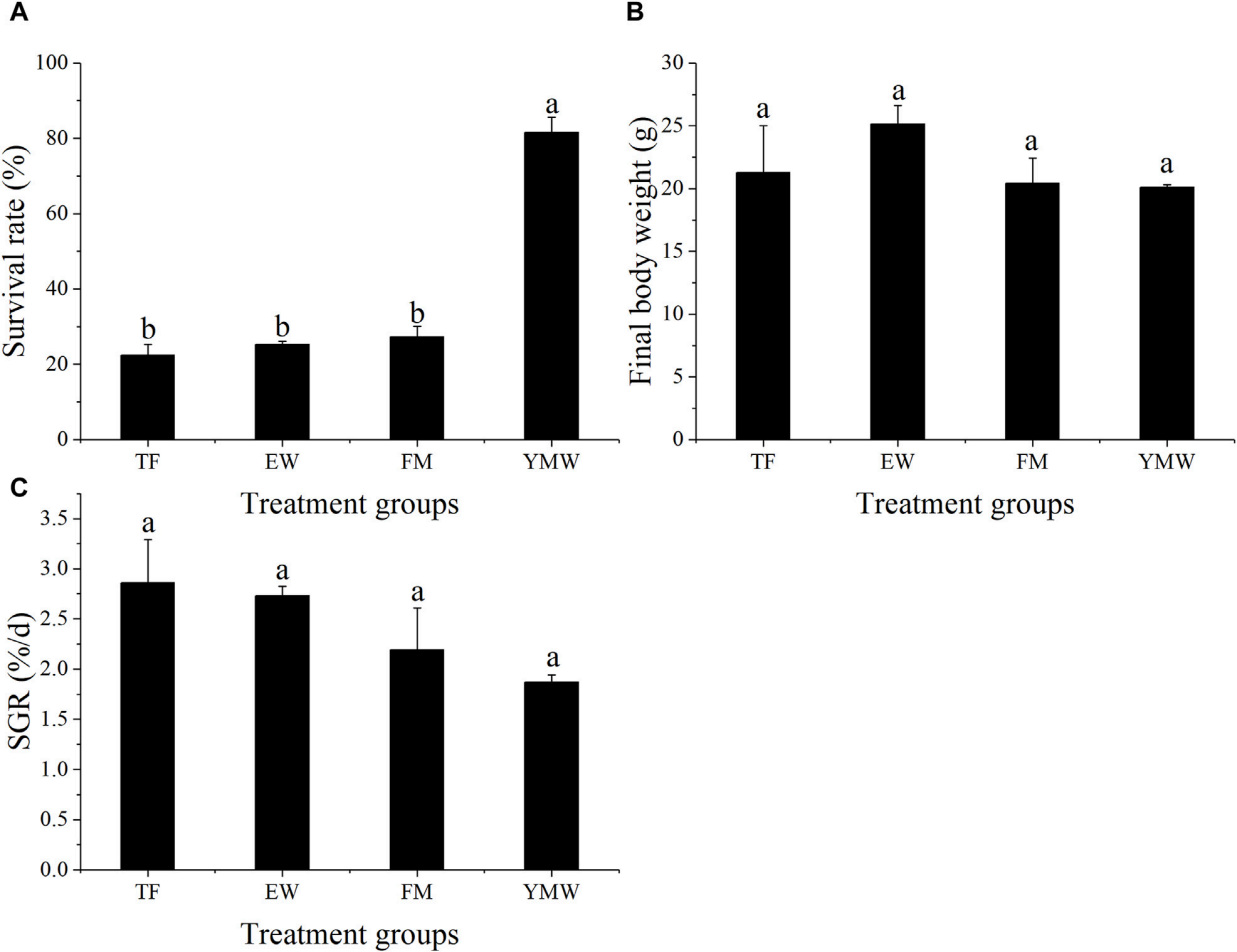
Survival rate **(A)**, final body weight **(B)**, and SGR **(C)** of four bait groups. Different letters in the column indicate significant differences between bait groups (*p* < 0.05). TF, Trash fish; EW, Earthworm; FM, Fly maggot; YMW, yellow mealworm.

The survival rate and SGR of the different bait groups are provided in Figure 1. The survival rate of the YMW group was significantly higher than that of the other three bait groups (Figure 1A, *p* < 0.05), with no significant differences in the survival rates of *M. albus* of the other three groups (*p* > 0.05). The survival rate of the YMW group was 73.33%–85.33%, and the survival rates of the other bait groups were less than 40%.

No significant differences were observed in the final body weight and SGR of *M. albus* among the four bait groups (Figures 1B, C, *p* > 0.05). The EW group showed the highest final body weight, with an average SGR of 2.73, whereas the YMW group had an average SGR of 1.87.

### 3.2 Feeding characteristics

**FIGURE 2:**
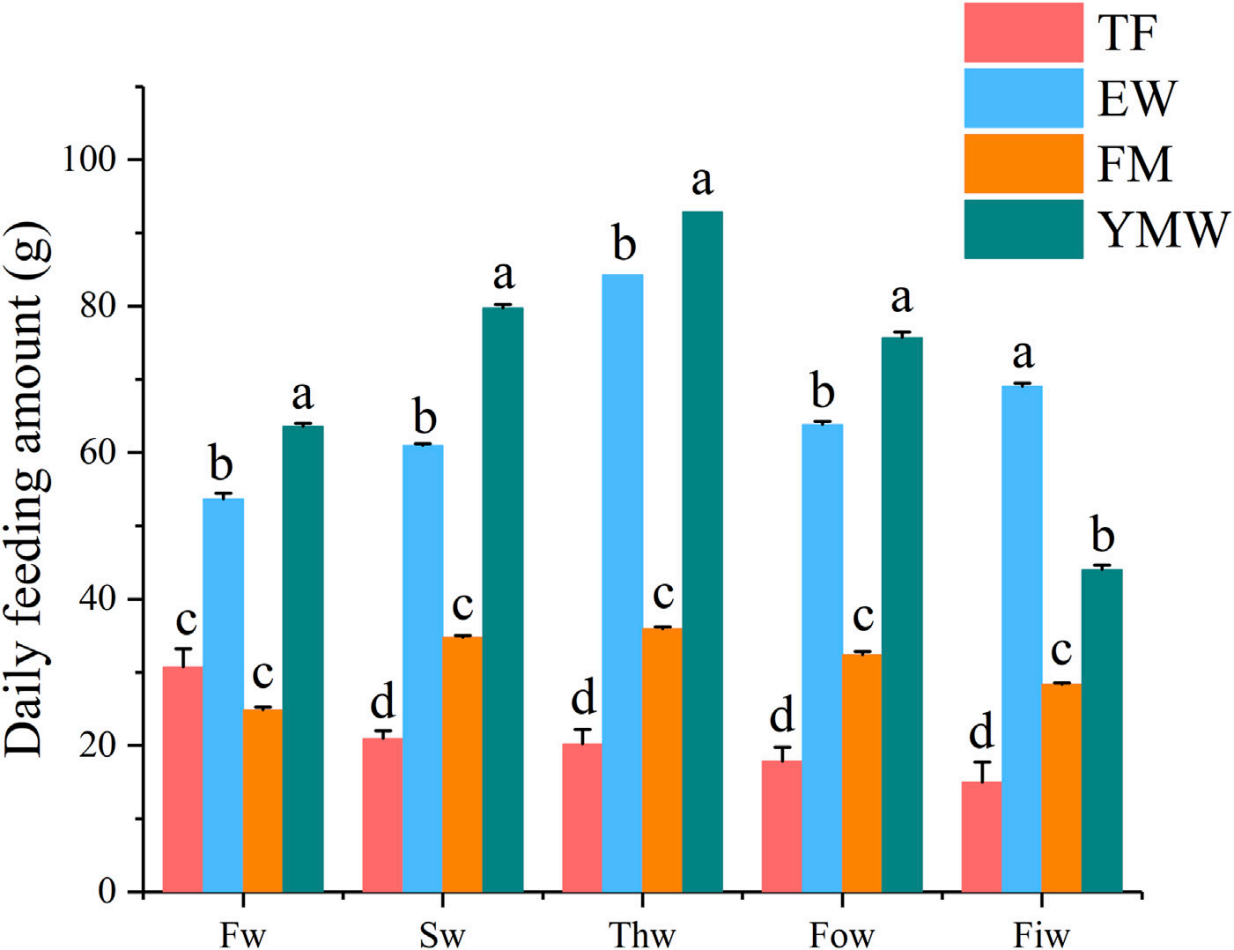
Daily feeding amount per week of four bait groups. Different letters in the column indicate significant differences between bait groups (*p* < 0.05). Fw, first week; Sw, second week; Thw, third week; Fow, fourth week; Fiw, fifth week. TF, Trash fish; EW, Earthworm; FM, Fly maggot; YMW, yellow mealworm.

A summary of the feeding data is presented in Table 2, and the average daily feeding amounts per week of the different bait groups are provided in Figure 2. A significant difference in feeding amounts was found between the different bait groups (*p* < 0.05). The total feeding amount and daily average feeding amount were significantly higher in the YMW group than in the other bait groups, followed closely by the EW group. The percentage of feeding amount to fish weight in the four bait groups was as follows: TF group, 2.14%–2.17%; EW group, 3.61%–7.3%; FM group, 1.72%–3.58%; and YMW group, 1.79%–3.98% (Table 2). Unlike the other three groups, the percentage of feeding amount to fish weight in the YMW group decreased with the extension of culture time. The average daily feeding amount was significantly higher in the EW and YMW groups than in the other two groups (*p* < 0.05; Figure 2). The percentage of feeding amount to fish weight in the EW group reached 7.3% in the fifth week, indicating the best feeding effect.

**TABLE 2:** Summary statistics for feeding-related parameters of *M. albus* in four bait groups. TF, Trash fish; EW, Earthworm; FM, Fly maggot; YMW, yellow mealworm.

Treatment group	Total feeding amount (g)	Daily feeding amount (g)	Feeding percentage (%)	
			First week	Fifth week
TF	716.67 ± 63.59^d^	21.07 ± 1.87^d^	2.14 ± 0.39^b^	2.17 ± 0.32^b^
EW	2,262.33 ± 12.72^b^	66.54 ± 0.37^b^	3.61 ± 0.13^a^	7.3 ± 0.58^a^
FM	1,064.00 ± 7.63^c^	31.29 ± 0.22^c^	1.72 ± 0.08^b^	3.58 ± 0.64^b^
YMW	2,431.67 ± 13.01^a^	71.52 ± 0.38^a^	3.98 ± 0.12^a^	1.79 ± 0.06^b^

Note: Values with different superscripts in each column are significantly different (*p* < 0.05).

Feeding percentage (%) of first week = daily feeding amount of first week (g)/initial body weight (g) × 100%.

Feeding percentage (%) of fifth week = daily feeding amount of fifth week (g)/final body weight (g) × 100%.

### 3.3 Water quality

**FIGURE 3:**
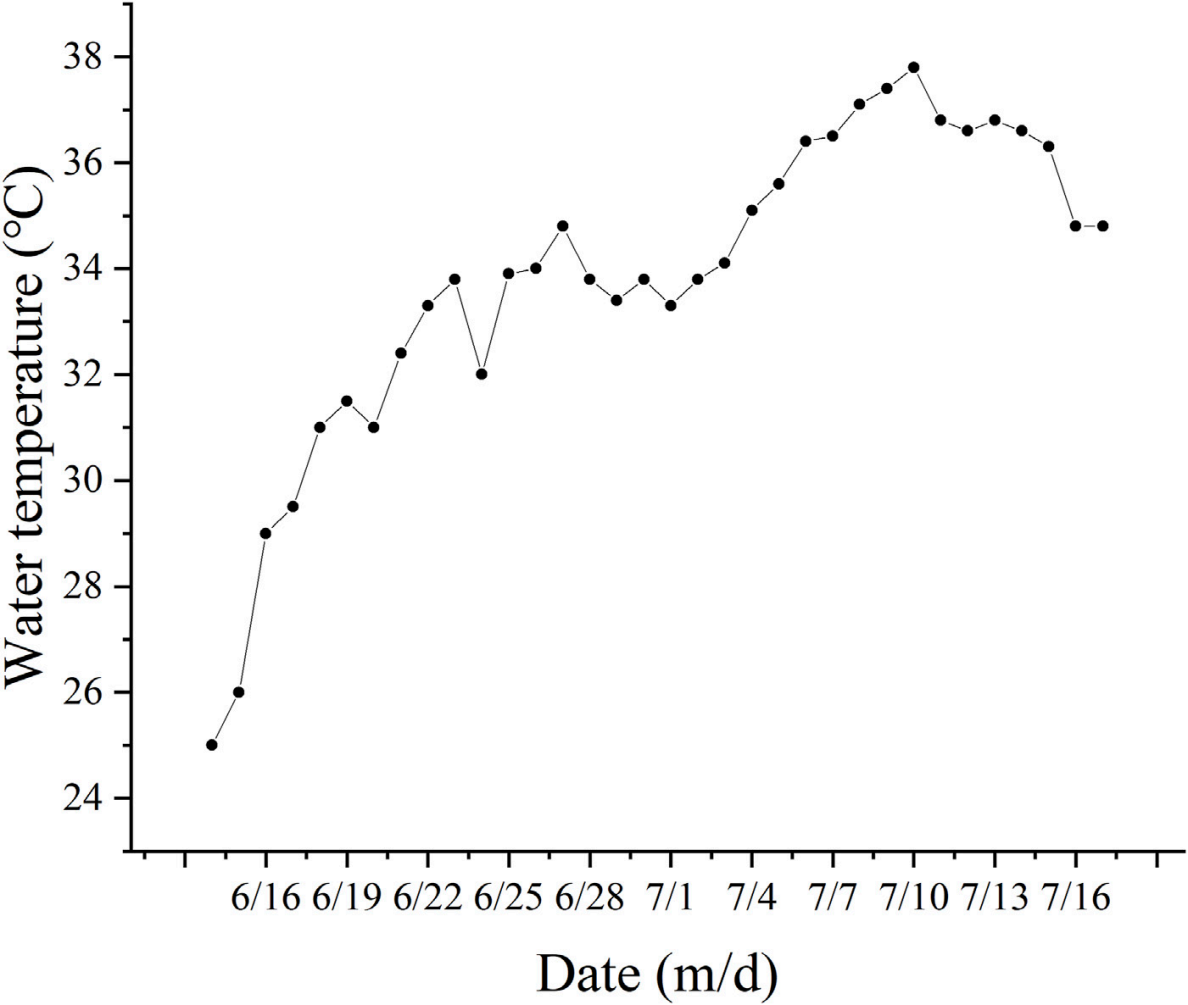
Changes in water temperature during the experiment.

**FIGURE 4:**
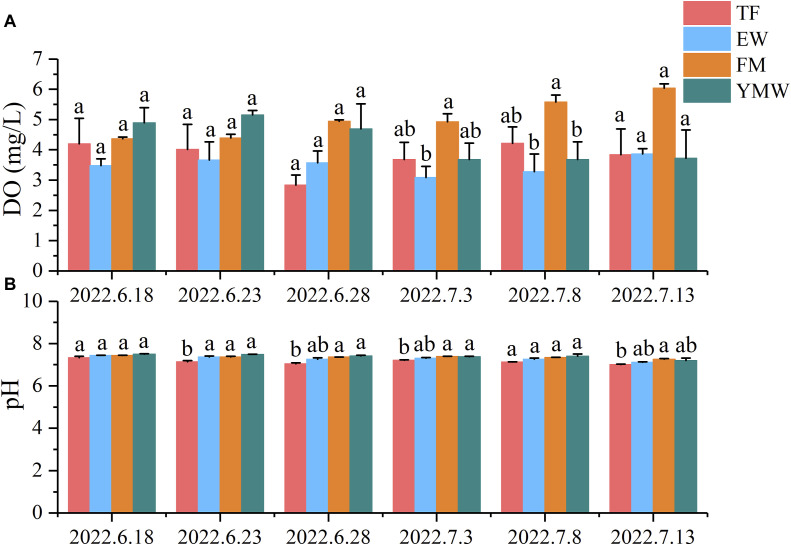
Changes in dissolved oxygen **(A)** and pH **(B)** of water in four bait groups. Different letters in the column indicate significant differences between bait groups (*p* < 0.05). TF, Trash fish; EW, Earthworm; FM, Fly maggot; YMW, yellow mealworm.

During the experiment, the range of water temperature variation was 25°C–36.8°C (Figure 3). The average dissolved oxygen (DO) concentrations in the TF groups ranged from 2.83 to 4.21 mg/L, while those in the EW group ranged from 3.08 to 3.86 mg/L. In the FM groups, the average DO concentrations ranged from 4.36 to 6.04 mg/L, and in the YMW groups, it ranged from 3.68 to 5.15 mg/L. There was no significant difference in the average DO concentrations among the treatment groups during the first three monitoring periods (Figure 4A, *p* > 0.05). Conversely, the FM group’s DO concentrations increased with extended aquaculture duration. The pH levels of the water across various bait groups ranged from 6.95 to 7.63 (Figure 4B). During the second, third, and fourth monitoring sessions, the pH value of the TF group was significantly lower than those of the FM and YMW groups (*p* < 0.05).

**FIGURE 5:**
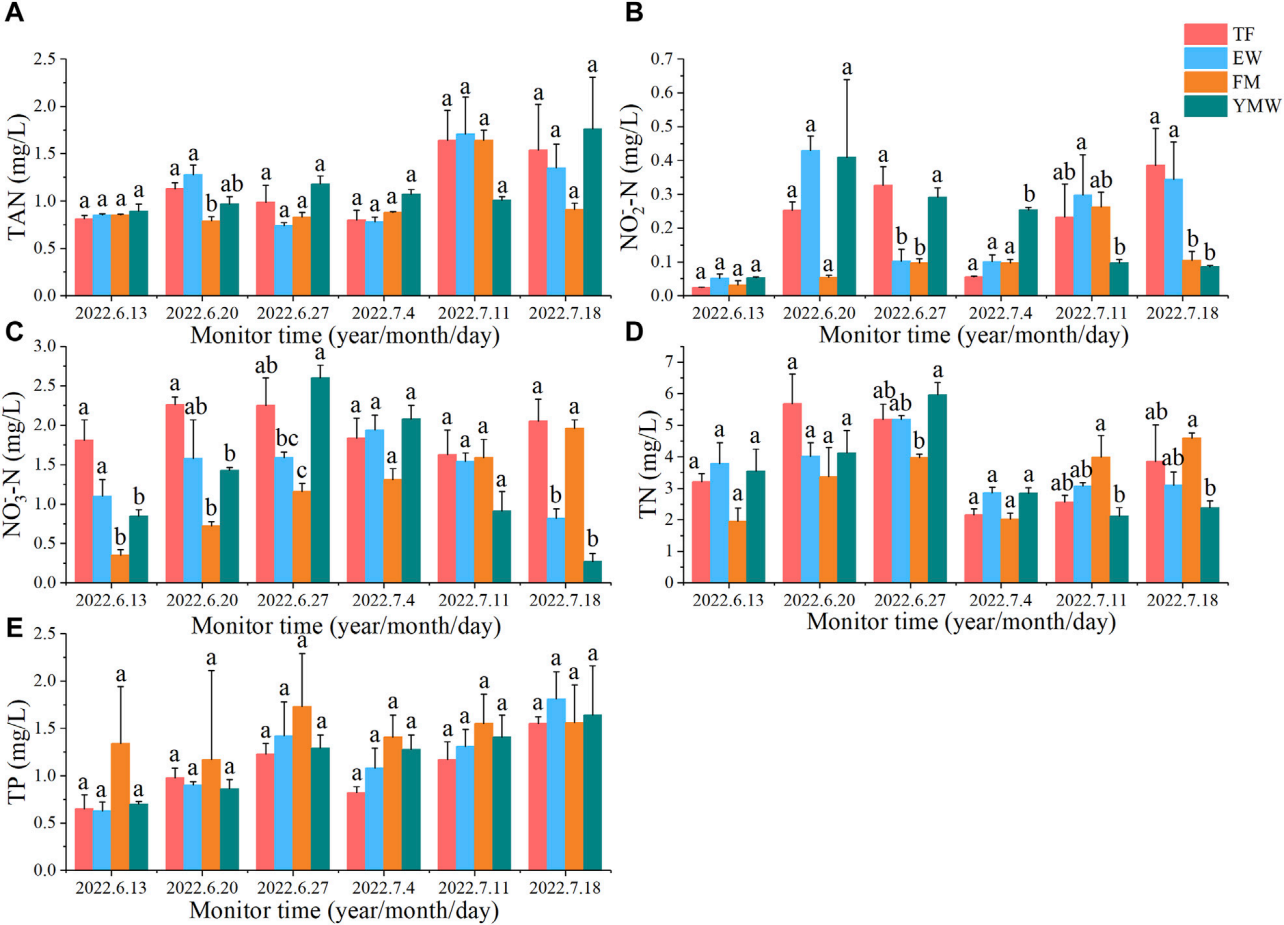
Changes in water quality indicators of four bait groups. Different letters in the column indicate significant differences between bait groups (*p* < 0.05). TAN, total ammonia nitrogen **(A)**; NO_2_^−^-N, nitrite nitrogen **(B)**; NO_3_^−^-N, nitrate nitrogen **(C)**; TN, total nitrogen **(D)** TP, total phosphorus **(E)**. TF, Trash fish; EW, Earthworm; FM, Fly maggot; YMW, yellow mealworm.

Except for the second monitoring session, there was no significant difference in TAN content among the treatment groups during the other monitoring sessions. (*p* > 0.05). After 1 weeks of cultivation, the average TAN content in the water was significantly lower in the FM group than in the TF and EW groups (*p* < 0.05). After 5 weeks of aquaculture, the average TAN content in the water significantly increased in the YMW group, reaching an average of 1.76 mg/L (Figure 5A).

NO_2_^−^-N content in the TF group peaked after 4 weeks of cultivation, whereas that in the YMW group peaked (0.41 mg/L) after 1 week of cultivation. After 5 weeks of aquaculture, NO_2_^−^-N content was significantly higher in the waters of the TF and EW groups than in the waters of the FM and YMW groups (*p* < 0.05, Figure 5B).

The NO_3_^−^-N content in the EW group peaked (1.94 mg/L) after 3 weeks of cultivation, whereas that in the YMW group peaked (2.60 mg/L) after 2 weeks of cultivation. After 5 weeks of aquaculture, the NO_3_^−^-N content was significantly higher in TF and FM groups than in EW and YMW groups (*p* < 0.05, Figure 5C).

There was no significant difference in TN content among the treatment groups both before the experiment and 1 week after the aquaculture trial (*p* > 0.05). After four and 5 weeks of cultivation, the average TN content was significantly higher in the FM group than in the YMW group (*p* < 0.05). After 3 weeks of aquaculture, the average TN content significantly decreased in the water bodies of each bait group, with no significant differences between the bait groups in the third week (*p* > 0.05). With the extension of time, TN content gradually increased in the TF and FM groups, whereas TN content showed no significant changes in the EW and YMW groups (Figure 5D).

The TP content in the water of each bait group first increased and then decreased over aquaculture time and began to decrease after 2 weeks of aquaculture, similar to the changes in TN content. Throughout all monitoring sessions, there was no significant difference in TP content among the treatment groups (Figure 5E, *p* > 0.05).

### 3.4 Antioxidant enzyme activities

**FIGURE 6:**
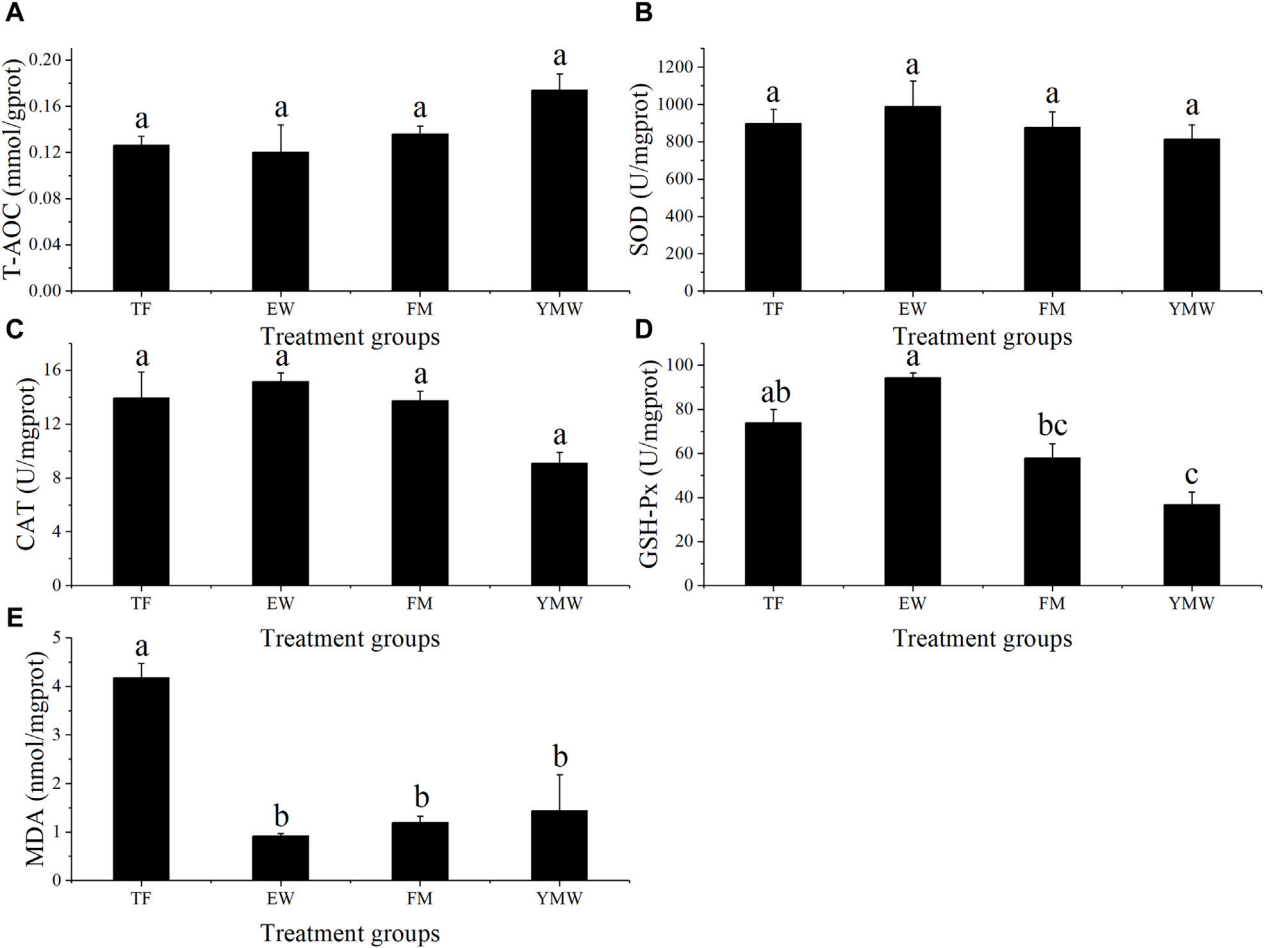
Changes in antioxidant enzyme activities of *M. albus* in four bait groups. Different letters in the column indicate significant differences between bait groups (*p* < 0.05). T-AOC, total antioxidant capacity **(A)**; SOD, superoxide dismutase **(B)**; CAT, catalase **(C)**; GSH-Px, glutathione peroxidase **(D)**; MDA, malondialdehyde **(E)**. TF, Trash fish; EW, Earthworm; FM, Fly maggot; YMW, yellow mealworm.

Changes in antioxidant enzyme activities in the liver of *M. albus* in each group are shown in Figure 6. There was no significant difference in T-AOC among the treatment groups (Figure 6A, *p* > 0.05). No significant differences in SOD and CAT activity were observed among the bait groups (Figures 6B, C, *p* > 0.05). GSH-Px activity was significantly lower in the YMW group than in the TF and EW groups (*p* < 0.05), whereas GSH-Px activity was significantly higher in the EW group than in the FM group (Figure 6D, *p* < 0.05). MDA content was significantly higher in the TF group than in the other bait groups (*p* < 0.05), with no significant differences among the other three groups (Figure 6E, *p* > 0.05).

### 3.5 Body composition

At the beginning of the experiment, no significant differences were observed in the nutritional composition indicators of *M. albus* among the bait groups (*p* > 0.05). After 5 weeks of cultivation, there were no significant differences in the composition indices of *M. albus* among the bait groups (*p* > 0.05).

After 5 weeks of farming with different bait types, the moisture content of *M. albus* decreased. The TF group showed a decrease in crude ash content, whereas the other three groups showed a significant increase in crude ash content. The crude fat and crude protein contents of the four bait groups significantly increased, with no significant differences in the rate of increase in crude fat content among the bait groups. The rate of increase in crude protein content was significantly higher in the TF group than in FM groups (*p* < 0.05, Table 3).

**TABLE 3:** Whole-body composition of *M. albus*. TF, Trash fish; EW, Earthworm; FM, Fly maggot; YMW, yellow mealworm.

Index	Treatment group	*M. albus* at the beginning of the experiment	*M. albus* at the end of the experiment	Increase in percentage (%)
Moisture content (%)	TF	64.15 ± 3.24	62.52 ± 0.56	−7.19 ± 1.72
	EW	69.78 ± 1.70	68.26 ± 0.23	−4.32 ± 1.79
	FM	68.88 ± 0.53	66.52 ± 0.99	−3.44 ± 0.98
	YMW	69.64 ± 1.10	67.75 ± 1.42	−2.70 ± 1.79
Crude protein (%)	TF	15.72 ± 0.08	21.26 ± 0.16	35.18 ± 0.45^a^
	EW	16.29 ± 0.24	18.97 ± 0.72	16.17 ± 6.03^ab^
	FM	17.30 ± 0.42	19.52 ± 0.48	12.89 ± 2.24^b^
	YMW	15.87 ± 0.57	19.95 ± 0.89	25.61 ± 2.40^ab^
Crude fat (g/100 g)	TF	3.99 ± 0.45	5.85 ± 0.41	48.19 ± 6.86
	EW	5.11 ± 0.41	6.09 ± 0.01	25.09 ± 16.36
	FM	4.59 ± 0.26	6.23 ± 0.35	36.81 ± 10.59
	YMW	4.46 ± 0.46	6.64 ± 0.65	50.31 ± 8.35
Crude ash (g/100 g)	TF	4.29 ± 0.67	3.21 ± 0.37	−23.47 ± 7.05^c^
	EW	3.93 ± 0.19	4.17 ± 0.04	9.91 ± 8.68^ab^
	FM	3.45 ± 0.55	5.05 ± 0.28	54.79 ± 27.02^a^
	YMW	3.16 ± 0.86	3.19 ± 0.32	18.55 ± 41.58^bc^

Note: Values with different superscripts in each column are significantly different for each index (*p* < 0.05).

## 4 Discussions

Recently, there has been a widespread increase in research on the applications of insect protein, especially from house flies and yellow mealworms, as a substitute for fish meal in aquaculture. Most of the studies have reported processes by which insects were converted into powder and added to feed, and few studies analyzed the effects of feeding live insect bait to fish (Maulu et al., 2022). *M. albus* prefers to eat live bait, and studying the effects of different insect larvae on their feeding and growth is of great significance for practical production.

The ANOVA results showed that recirculating aquaculture with four different bait types had significant effects on the survival rate of *M. albus*. The highest survival rate was found in the YMW group and the lowest in the TF group, presumably because the water quality was suitable or at least in the feasible range to support the maintenance of *M. albus*. This can be observed from the changes in TAN and NO_2_^−^-N indicators. TAN and NO_2_^−^-N are environmental factors that are more likely than other environmental factors such as NO_3_^−^-N to exert stress on fish (Portalia et al., 2019). In this study, the DO content in the TF and EW groups was significantly lower than that in the FM and YMW groups, and the pH levels were also lower than those in the FM and YMW groups, consistent with the general trend of a positive correlation between DO and pH levels. The pH level primarily fluctuates due to CO_2_ dynamics, influenced by factors such as algae photosynthesis, aquatic organisms’ respiration, and the oxidation and decomposition of organic matter, as CO_2_ is a byproduct of organic decay. It is widely acknowledged that aquatic plants absorb CO_2_ during photosynthesis, leading to a rise in pH levels, the production of oxygen, and an increase in DO concentrations (Yuan et al., 2023). The TN, TP, and NO_3_^−^-N content in the recirculating aquaculture water remained within normal ranges overall. Between July 4th and 11th, the water temperature rapidly increased from 33°C to 37°C, whereas TAN content fluctuated less in the YMW group and rapidly increased in the other groups, exceeding 1.5 mg/L. This may be because YMW is a live bait, and, even if it was available in surplus, it decomposes slowly in the water, while the other bait types were all frozen. Although TAN content in the YMW group increased significantly in the last week of the experiment, this may be related to the continuous cultivation of high-stock fish, whereas TAN content in the other groups decreased because of more *M. albus* deaths, resulting in a decrease in feeding volume and TAN content in the last week. The toxicity of ammonia nitrogen to cultured animals is closely related to temperature and pH values. With higher pH and temperature, the proportion of free ammonia (NH_3_) increases, leading to a significant increase in toxicity. In this study, the average pH values of the four treatment groups ranged from 7.0 to 7.5, with most of the rearing period experiencing water temperatures exceeding 30°C. Under these conditions, the proportion of free ammonia accounted for approximately 1% of the total ammonia concentration (Zhu et al., 2021). The Asian swamp eel is normally considered an air-breathing fish capable of lowering the toxicity of ammonia in their environment and body via unique strategies of ammonia detoxification (Ip et al., 2004; Chew et al., 2005; Chen et al., 2013); hence, the ammonia nitrogen content in present study should not exert direct stress on the *M. albus*. In addition, NO_2_^−^-N content in the YMW group decreased continuously; the significant increase after 1 week of aquaculture may be related to the recirculating water treatment system, as biological filters require some time for membrane formation. However, simultaneous occurrences of elevated concentrations of ammonia nitrogen, high water temperatures, and insufficient DO indicate deteriorating water quality, which can easily lead to infections by opportunistic pathogenic bacteria, ultimately resulting in fish morbidity and mortality (Zaheen et al., 2022). This observation aligns with the phenomena we observed during the experimental process, where dead Asian swamp eels exhibited symptoms of bacterial infections such as external hemorrhaging or anal bleeding.

Another important reason for the high survival rate of *M. albus* may be the presence of antimicrobial peptide activity in YMW, which in turn enhances the immune system of *M. albus*. According to Xiang et al. (2019), insect meal contains bioactive peptides that may prevent diseases. Therefore, insect meal presents the potential prevention of intestinal inflammation in aquaculture. Incorporation of YMW in yellow croaker (*Larimichthys crocea*) diets in which fishmeal was replaced by 426.2–568.3 g/kg (75%–100% fishmeal replacement) led to increased muscle hardness and significantly lower shear force in fillets (Yuan et al., 2022). Replacing fishmeal with YMW in the diets of *L. vannamei* juveniles improved the survival rates of the shrimp after being challenged with the pathogenic bacterium *Vibrio parahaemolyticus* (Motte et al., 2019). In *P. fulvidraco*, 18% dietary YMW improved the immune response and disease resistance of the fish against the bacterium *Edwardsiella ictaluri* (Su et al., 2017). In juvenile mandarin fish (*Siniperca scherzeri*), the inclusion of YMW in the diets enhanced the immune system of the fish (Sankian et al., 2018). According to Motte et al. (2019), replacing fishmeal with 50% defatted YMW improved the disease resistance of *L. vannamei* against early mortality syndrome. In red seabream (*Pargus major*), feeding the fish with diets containing YMW after challenge with a bacterial pathogen (*Edwardsiella tarda*) improved survival (Ido et al., 2019). Henry et al. (2018) used YMW to feed the rainbow trout (*Oncorhynchus mykiss*) for 6 weeks. The fish showed a significant enhancement in anti-inflammatory responses, lysozyme antibacterial activity, and serum trypsin inhibition, which are usually related to anti-parasitic activity. These findings show that insect meal may help to improve disease resistance in fish farming.

No significant differences in SGR of *M. albus* were detected among the bait groups, but the YMW group had the lowest SGR. Because the YMW group showed the highest survival rate, a decrease in SGR may have been caused by the relatively high-density recirculating aquaculture. This is in accordance with the findings of Yuan et al. (2018), who stated that stocking density will affect the competition for space. Limited space for movement will cause *M. albus* to become more easily stressed, which will then affect growth. The causes for the slowed growth of fish are a large level of competition between individuals for food, space for movement, oxygen consumption, and the amount of waste material, including carbohydrates, ammonia and nitrite, in the water that can interfere with the fish (Portalia et al., 2019). In addition, growth rate and nutrient quality of *M. albus* in freshwater aquaculture are influenced by natural feed (Herawati et al., 2018). Different natural feed intakes may also affect the SGR of *M. albus*, but this needs to be studied further by conducting longer culture experiments.

For *M. albus*, multiple low-dose feedings were performed, so the feeding amount was closer to the food intake amount. In this study, significant differences in feeding amount were found among the different groups, with the YMW group having the highest feeding amount, followed by the EW group. The EW group had the highest feeding percentage; the YMW group had a higher feeding percentage in the first week, but it decreased with time. This indicates that *M. albus* prefers to eat yellow mealworms and earthworms more than fly maggots and trash fish. *M. albus* are responsive to live baits such as aquatic invertebrates, small fish, and crustaceans with nocturnal foraging behavior prevailing (Hill and Watson, 2007). Live baits can elicit a strong feeding response due to their natural movement and scent, making them effective for angling and aquaculture purposes. This indicates that live bait such as yellow mealworms is more conducive to training Asian swamp eels to accept feeding compared to frozen baits.

After 40 days of feeding with different natural bait types, the crude protein and fat contents of the whole body of *M. albus* significantly increased. The TF group showed the highest rate of increase in crude protein content, followed by the YMW group. This indicates that the utilization efficiency of *M. albus* for trash fish protein is higher than that for insect protein, but the specific reasons need to be evaluated. At the end of the experiment, the crude protein content of *M. albus* was higher than that of *M. albus* cultivated with a formulated diet (Ma et al., 2014; Yue et al., 2021). Ma et al. (2014) found that, after 10 weeks of mixed feed cultivation, the highest crude protein content of *M. albus* was 16.78%. The moisture content of *M. albus* in this study was significantly lower than that of *M. albus* cultured with a formulated diet (more than 70%) (Ma et al., 2014; Yue et al., 2021).

Aerobic metabolism has the potential to generate reactive oxygen species (free radicals), whose elevated levels can induce oxidative damage, leading to oxidative stress (Kakade et al., 2020). SOD, CAT, GSH-Px, T-AOC, and MDA serve as crucial markers of antioxidant activity. SOD and CAT function by scavenging free radicals, mitigating oxidative stress-induced damage to the intestinal mucosa, thereby playing a pivotal role in intestinal defense and repair. GSH-Px reflects the organism’s capability to neutralize free radicals, safeguarding against lipid peroxide-induced harm. T-AOC serves as a comprehensive indicator of antioxidant capacity, illustrating the organism’s ability to counteract external stressors and the status of free radical metabolism. MDA content indicates the extent of lipid peroxidation and indirectly reflects cellular damage severity (Wang et al., 2022). In this study, the MDA content in the liver of the TF group was significantly higher than that of the other bait groups, which implies that insect bait can reduce lipid peroxidation and oxidative stress. Numerous studies have shown that housefly maggots have positive effects on the growth performance of various farmed animals (Yi et al., 2014; Xiang et al., 2019). Among the insect feed groups, liver GSH-Px activities was significantly higher in the EW group than in the YMW group, with no significant differences when compared with the FM group. Many studies have reported the feeding and growth of fish with earthworms (Parolini et al., 2020). Earthworm dry matter (16%–20% of fresh matter) contains from 55% to 70% proteins (Mohanta et al., 2016) and earthworms are easy to cultivate, making them a promising alternative protein in fish farming (Parolini et al., 2020). This study shows that earthworms may be beneficial for enhancing the antioxidant stress capacity of *M. albus*.

Currently, the unit price of yellow mealworm larvae available in the Chinese market ranges from 12 to 16 RMB/kg, frozen earthworms are priced at 8 to 10 RMB/kg, frozen fly maggots at 4 RMB/kg, and frozen trash fish at 5 to 6 RMB/kg. Yellow mealworms are insects with a fast growth cycle and are easy to rear. They can be raised on balconies or in old buildings, making them a cost-effective option for bait production, with bait costs potentially controlled below 8 RMB/kg through supplementary breeding. Considering the high survival rate of Asian swamp eels trained to accept yellow mealworms as indicated in present study, and with the continuous decrease in natural resources of trash fish (Tacon and Metian, 2008), using yellow mealworms as an alternative bait option demonstrates significant economic feasibility. However, natural insect baits, particularly yellow mealworms, due to their high lipid content, may pose adverse health effects on cultured animals with prolonged and extensive usage. Therefore, further research is needed to develop feeding strategies that not only does it domesticate Asian swamp eels with natural baits for feeding, but also promote their long-term health and growth.

## 5 Conclusion

According to the results of this study, aquaculture of *M. albus* with natural insect bait rather than trash fish has significant advantages in terms of survival rate, water quality. The YMW group exhibited the highest survival rate during recirculating aquaculture, relatively good water quality. Therefore, *T. molitor* could potentially serve as one of the alternative feeds during the initial stages of *M. albus* juveniles stocking. The second-best natural bait is earthworms, which has significant advantages with respect to feeding rate and antioxidant enzyme activity of *M. albus*.

## Data Availability

The original contributions presented in the study are included in the article/Supplementary material, further inquiries can be directed to the corresponding author.
